# Supplementary figures and images for: Luteolin target HSPB1 regulates endothelial cell ferroptosis to protect against radiation vascular injury
Source: PLoS One. 2024 Oct 11;19(10):e0311922. doi: 10.1371/journal.pone.0311922 (PMC11469493; doi:10.1371/journal.pone.0311922)

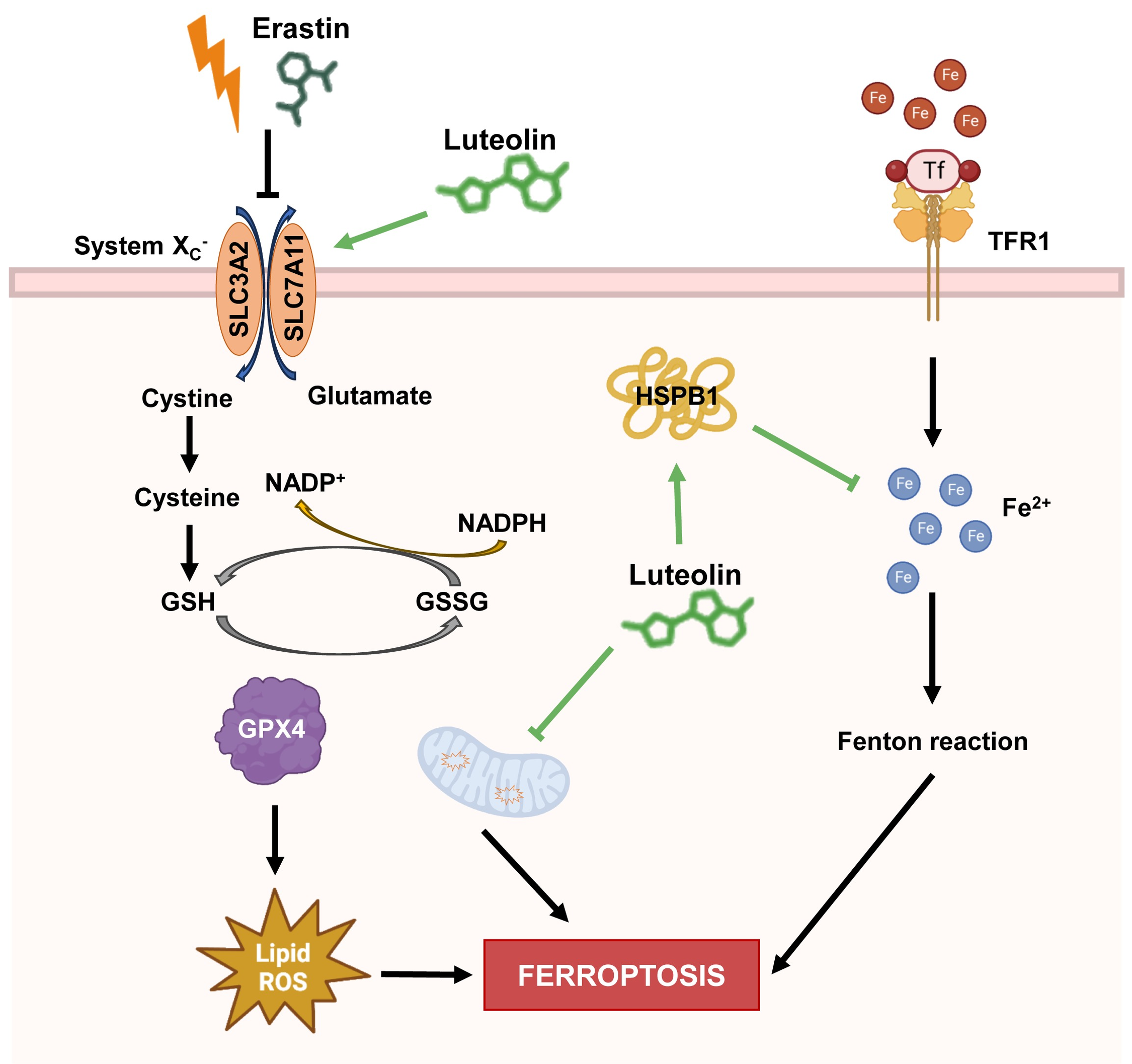

Supplement: S1 Graphical abstract — (TIF) [file pone.0311922.s001.tif]
