# Supplementary material for: Luteolin target HSPB1 regulates endothelial cell ferroptosis to protect against radiation vascular injury
Source: PLoS One. 2024 Oct 11;19(10):e0311922. doi: 10.1371/journal.pone.0311922 (PMC11469493; doi:10.1371/journal.pone.0311922)

Fig 5C

Input-DMSO; Input-Luteolin; protease1:1000-DMSO; protease1:1000- Luteolin; protease1:2000-DMSO; protease1:2000- Luteolin

HSPB1

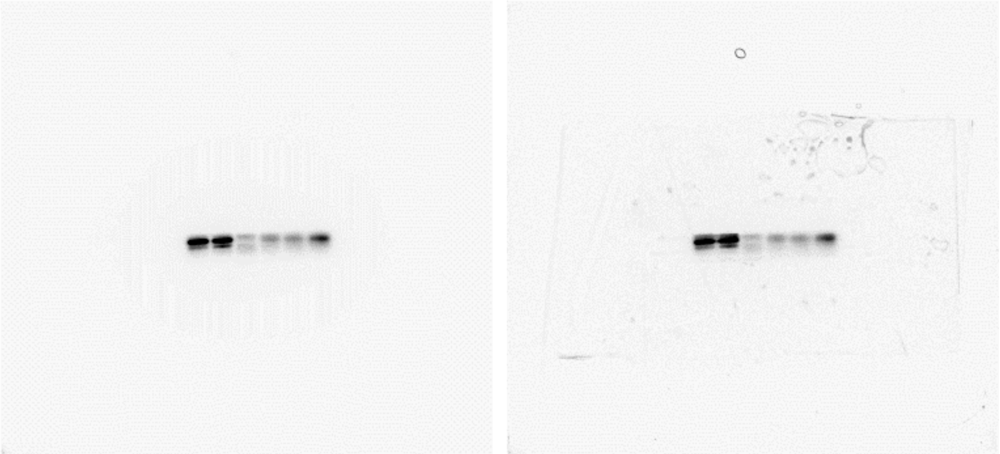

GAPDH

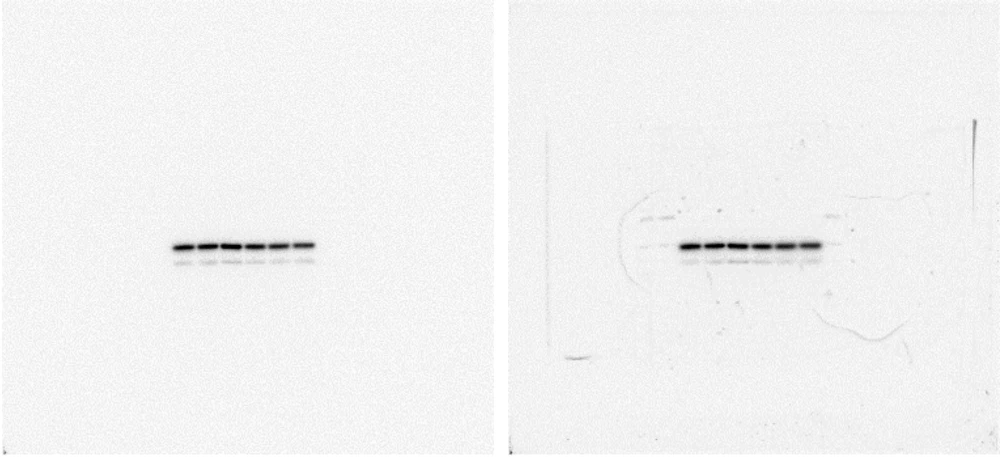

Fig 5D

Luteolin-0μM;5μM;10μM;20μM;40μM

GAPDH

HSPB1

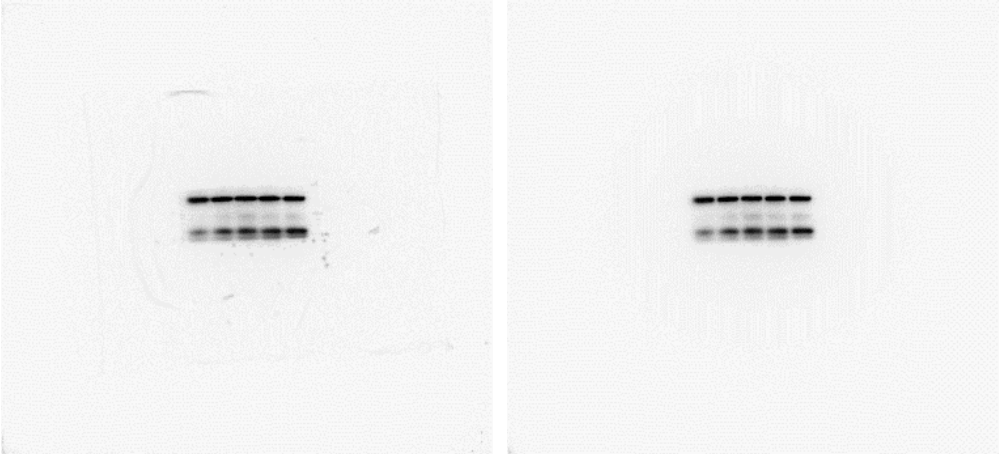

Fig 5E

Control; erastin; erastin+luteolin; erastin+fer-1

SLC7A11

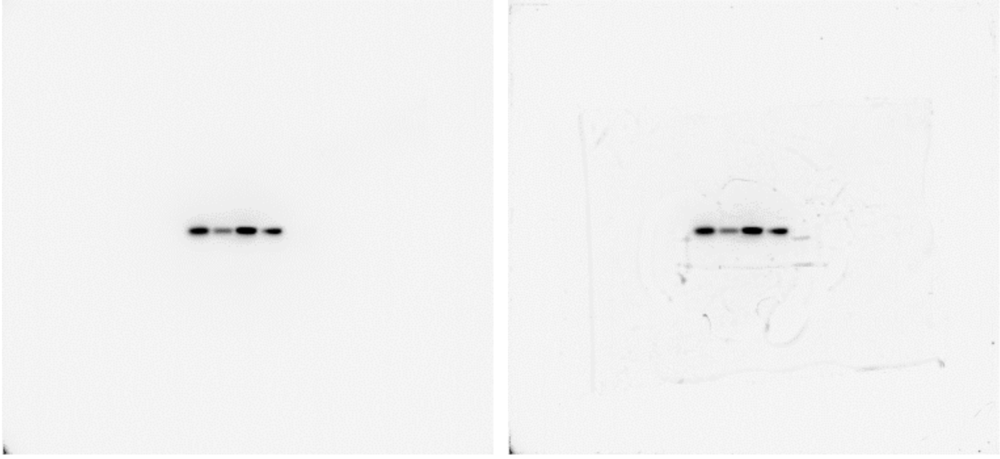

GPX4

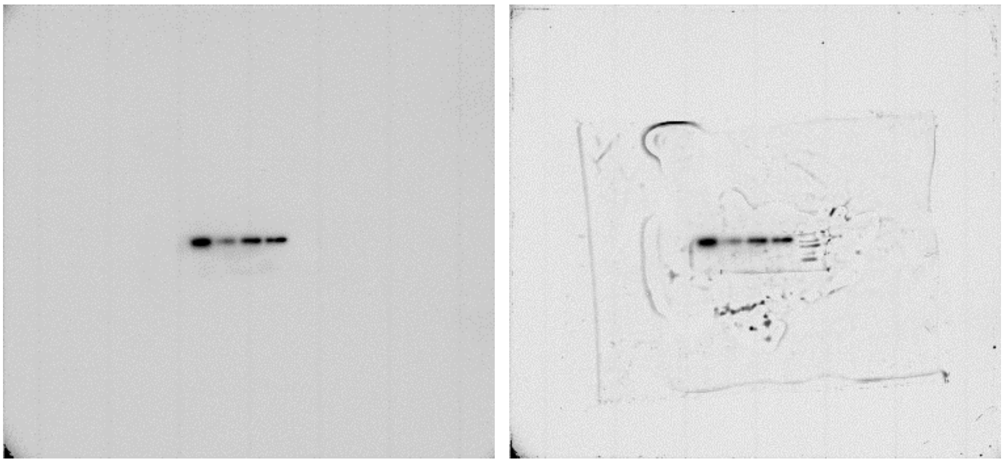

GAPDH+HSPB1

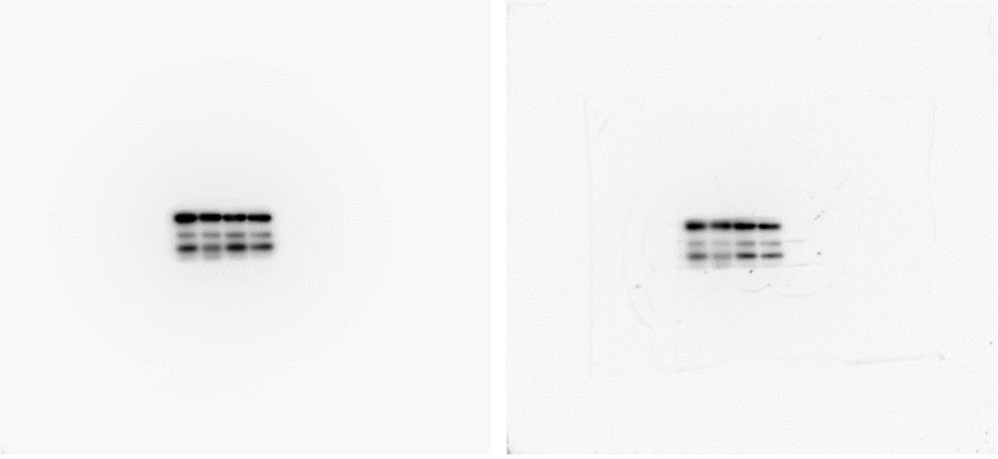

Supplement: S1 File — (PDF) [file pone.0311922.s002.pdf]
